# Effectiveness and safety of cyclosporine A in moderate to severe COVID-19: a randomized, open-label trial

**DOI:** 10.1038/s41598-026-35292-0

**Published:** 2026-02-17

**Authors:** Amira A. Zidan, Ahmed Y. S. Gad, Nermine H. Zakaria, Hazem M. El-Hariri, Noha M. Elsharnouby, Maged W. Helmy, Maged El-Setouhy

**Affiliations:** 1 Department of Clinical Pharmacy, El-Beheira Health Affairs, Ministry of Health, Damanhour, Egypt; 2 Department of Chest Disease, Faculty of Medicine, Alexandria University, Alexandria, Egypt; 3 Department of Clinical Pathology, Faculty of Medicine, Alexandria University, Alexandria, Egypt; 4 Department of Community Medicine, National Research Centre, Cairo, Egypt; 5 Department of Anesthesia, Intensive Care and Pain Management, Faculty of Medicine, Ain-Shams University, Cairo, Egypt; 6 Department of Pharmacology and Toxicology, Faculty of Pharmacy, Damanhour University, Damanhour, Egypt; 7 Department of Family and Community Medicine, Faculty of Medicine, Jazan University, Jazan, Kingdom of Saudi Arabia; 8 Department of Community, Environmental and Occupational Medicine, Faculty of Medicine, Ain Shams University, Cairo, Egypt

**Keywords:** Cyclosporine, Standard treatment, Interleukin-2 (IL-2), Interleukin-6 (IL-6), Covid-19, Hyperinflammatory markers, Hyperinflammation, Ferritin, D-dimer, C-reactive protein (CRP)

## Abstract

COVID-19 severity is strongly associated with hyperinflammation. Cyclosporine A (CSA), an interleukin-2 inhibitor with immunomodulatory and antiviral activity, has been proposed as a potential adjunctive therapy. This study evaluated the safety and efficacy of CSA in patients with moderate to severe COVID-19. We conducted A randomized, open-label phase III trial was conducted involving 66 patients with COVID-19. Participants were assigned to one of two groups: the CSA group (n = 23), receiving 6 mg/kg/day for 7–14 days, and a standard treatment group (n = 43). Clinical improvement (WHO ordinal scale) was the main goal, with C-reactive protein (CRP), ferritin, interleukin-6 ( IL-6), and D-dimer, and safety monitoring for 28 days as secondary outcomes significant differences in enrolment. The time to clinical improvement was significantly shorter in the CSA group (4.3 ± 1.0 vs. 5.1 ± 2.3 days; *p* = 0.025). Oxygen supplementation was used in 7 patients (30.43%) versus 12 patients (27.91%) in the standard group, with a *p*-value of 0.828. No significant differences occurred in the WHO ordinal scale, advanced respiratory support, or mortality. No secondary infections occurred. CSA improved oxygen saturation and reduced CRP and IL-6; differences in saturation at day 14 were not significant. D-dimer and ferritin levels were lower at day 14, with no differences observed at day 7. Cyclosporine did not significantly improve ordinal scale outcomes. However, it was associated with a shorter time to clinical improvement and favorable modulation of inflammatory markers in patients with COVID-19 and cytokine storm, without major safety concerns.

## Introduction

Coronaviruses were first identified in 1965^1^, including a novel strain that caused the global COVID-19 pandemic, leading to 7,077,725 deaths by December 1, 2024^2^. Severe COVID-19 is linked to a hyperinflammatory response similar to secondary hemophagocytic lymphohistiocytosis (sHLH). The disease can result in cytokine-mediated organ failure, presenting symptoms like fever, cytopenias, and hyperferritinemia, acute respiratory distress syndrome (ARDS)^3,4^. Additionally, viral infections may trigger hemophagocytic lymphohistiocytosis in genetically predisposed individuals^5^. Elevated cytokine levels, such as IL-2 and TNF-α, are characteristic of COVID-19 and can contribute to conditions like fulminant myocarditis and ARDS, with IL-2 potentially serving as an early marker for ARDS^6–8^.

Several studies have shown that cyclosporin (CSA) inhibits influenza virus replication through both cyclophilin A-dependent and independent pathways^9^. CSA has also shown antiviral effects in influenza A virus-infected mice^10^ and broadly inactivates coronaviruses, including SARS-CoV and several other strains^11–14^, as well as SARS-CoV-2^15,16^. Additionally, CSA exhibits antiviral activity against other viruses such as herpes simplex virus (HSV), vaccinia virus (VV)^17^, BK polyomavirus (BKV), HIV-1^18^, and hepatitis B virus^19^. It also shows strong efficacy against the hepatitis C virus (HCV)^20–22^.

In a 2020 review by Fung and Yuen, SARS-CoV-2 was found to be 82% genetically similar to SARS-CoV, with medications effective against SARS-CoV also showing activity against SARS-CoV-2^23^. Cyclosporine, known for its anti-inflammatory effects, blocks IFN-γ in T cells and halts cytokine gene transcription^16^, particularly interleukin-2 (IL-2), which may contribute to cytokine storms in COVID-19 patients. IL-2 has been identified as a candidate for early acute respiratory distress syndrome (ARDS) detection^8^. Henter’s studies from 1993 and 2007 noted that viral infections could trigger familial hemophagocytic lymphohistiocytosis in genetically predisposed individuals, with cyclosporine A being part of the treatment regimen^5^. Imashuku recommended short infusions of cyclosporine for Epstein–Barr virus-related HLH, combined with etoposide and corticosteroids, for better patient outcomes^24^. Studies suggest cyclosporine does not increase viral sensitivity and may be a suitable option during the COVID-19 pandemic, showing lower mortality risk and improved outcomes for kidney transplant recipients infected with COVID-19^25–28^.

Cytokine inhibitors through different mechanisms, such as IL-6 receptor blockade^29,30^, IL-1 blockade (anakinra)^31^, corticosteroids (general), methylprednisolone, and Janus kinase (JAK) inhibition, have been conducted^32^. However, there is little data on the efficacy of IL-2 inhibitors in the treatment of COVID-19. Cyclosporine A (CSA) is a known cytokine inhibitor that acts as an IL-2 inhibitor. It has demonstrated immunosuppressive effects since 1980 and was FDA-approved in 2000.

Several clinical trials have investigated the safety and efficacy of various medications, including Hydroxychloroquine,Favipiravir,Lopinavir and Ritonavir, Remdesivir^33,34^, Oseltamivir^35^, and Ivermectin^36^. While some studies suggest that these drugs may be effective, others indicate that they are not. We compared Cyclosporine with the protocols established by the Ministry of Health at the time of the study.

In this study, we aimed to evaluate the efficacy and safety of the IL-2 inhibitor cyclosporine compared to standard care, using a hospital-based treatment protocol for patients with COVID-19.

## Methodology

### Setting

This study was conducted at Alexandria University Hospital, one of the largest teaching hospitals in Egypt and the Mediterranean region, with a capacity of 4,500 beds. During the pandemic, it served as a primary referral center for patients with suspected COVID-19.

### Study design and participants

**Fig. 1:**
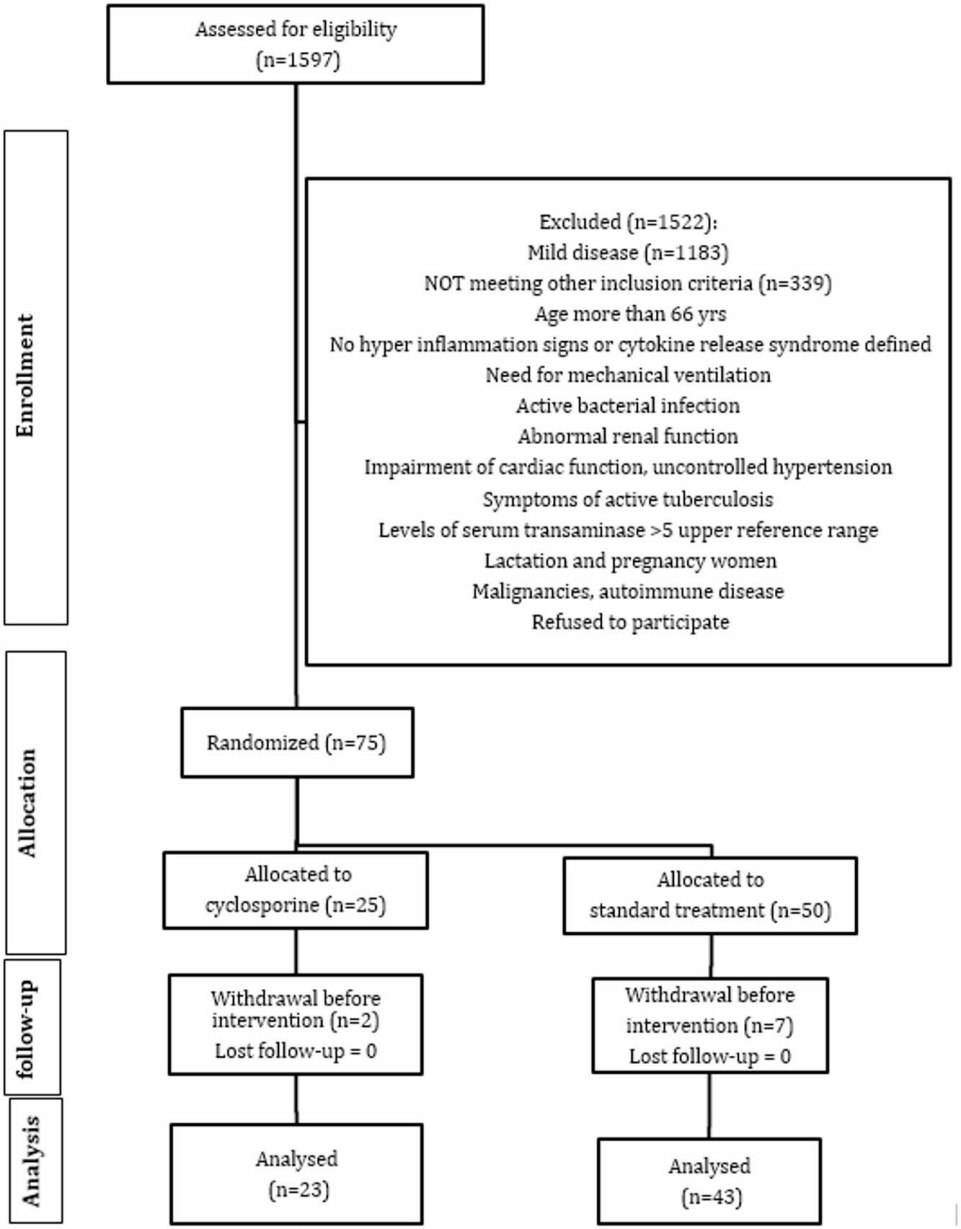
Flow chart of the studied cases.

Between January and September 2022, 1,597 adults with suspected COVID-19 were screened at Alexandria University Hospital using PCR-based nasopharyngeal testing. A comprehensive baseline evaluation, which included medical history, physical examination, and complete blood count, was collected. Seventy-five patients were randomized after meeting the inclusion criteria: age 18–65 years, a positive PCR test, evidence of hyperinflammation or cytokine release syndrome (e.g., leukopenia, lymphopenia, ferritin > 500 ng/mL, D-dimer ≥ 500 ng/mL, or low Hs90 levels)^37^, and having given written informed consent. After screening, eligibility criteria were applied, and patients were excluded if they were pregnant or lactating; intolerant to cyclosporine; had received monoclonal antibodies or live-attenuated vaccines within one week before admission; required mechanical ventilation at baseline; or had active bacterial or fungal infection, sepsis, or multi-organ failure. Additional exclusions included uncontrolled diabetes mellitus or hypertension, significant renal, hepatic, or cardiac dysfunction, gastrointestinal conditions such as perforation or diverticulitis, and use of systemic steroids > 20 mg methylprednisolone equivalent or other concurrent immunosuppressive therapy. Patients were also excluded if they were receiving methotrexate, other immunosuppressive agents, coal tar, radiation therapy, or biologics targeting IL-2, IL-6, or IL-1. Finally, those with active tuberculosis, autoimmune disease, HIV infection, or malignancy were not eligible (Fig. 1).

### Randomization and interventions

After randomization, all eligible patients underwent a comprehensive laboratory investigation, including complete blood count, ferritin, D-dimer, IL-6, C-reactive protein, renal and liver function tests, coagulation profile, electrolytes, and, where appropriate, a pregnancy test, Inflammatory risk was assessed using a composite index that integrates CRP, ferritin, IL-6, and D-dimer, based on standardized z-scores as previously described in the referenced resource^38–40^.

Patients were randomized in a 1:2 ratio using computer-generated sequences and sealed opaque envelopes.

A total of 25 patients were assigned to the cyclosporine group and 50 to the standard of care (SOC) group. After nine patients withdrew from the study before the intervention: seven from the SOC group and two from the cyclosporine group,23 patients in the cyclosporine group and 43 in the SOC group completed the study.

Cyclosporine group: Received cyclosporine 6 mg/kg/day in two divided doses for 8–14 days, with dose adjustments to maintain serum levels between 200–300 ng/mL^41–43^. All patients had normal kidney function. No antiviral agents were administered.

Control group: Received SOC according to the Egyptian Ministry of Health (2022).

Standard of care for both groups included:

Azithromycin 500 mg once daily for 5 days.

Ivermectin 6 mg (two tablets) on days 1, 3, and 6.

Antipyretics as needed.

Anticoagulation and any necessary supportive or therapeutic interventions were based on the patient’s clinical condition and the Ministry of Health’s treatment protocols.

### Follow-up and monitoring

Patients were monitored daily, either in hospital or remotely via telemedicine, for body temperature, oxygen saturation (measured by thermometer and pulse oximeter), and overall clinical status. Laboratory tests and SARS-CoV-2 PCR were repeated on Days 7 and 14.

Recovery was defined as: afebrile for ≥ 24 h without antipyretics, ferritin < 500 ng/mL, D-dimer < 500 ng/mL, and a negative PCR for SARS-CoV-2.

### Outcomes

The primary outcome was clinical status on a 7-point ordinal scale recommended by the World Health Organization(WHO), assessed at Days 7–14^44^:

Not hospitalized, no activity limitation. Not hospitalized, limited activity.

Hospitalized, no oxygen required. Hospitalized, requiring oxygen.

Hospitalized, requiring non-invasive ventilation/high-flow oxygen.

Hospitalized, requiring invasive ventilation. Death.

Secondary outcomes included:

Change in IL-6, ferritin, D-dimer, and CRP from baseline.

Incidence of grade 3–4 adverse events (per DAIDS v2.1).

Nosocomial bacterial or fungal infections within 28 days (confirmed by cultures and clinical findings). Mortality at 30 and 90 days.

### Statistical analysis

The collected data were coded, tabulated, and statistically analyzed using IBM SPSS Statistics (Statistical Package for Social Sciences) software, version 28.0, provided by IBM Corp., Chicago, USA, in 2021. Quantitative data were tested for normality using the Shapiro–Wilk test. They were then described as mean ± standard deviation (SD) and compared using independent t-tests and paired t-tests. Qualitative data were presented as numbers and percentages, and comparisons were made using the chi-square test and Fisher’s Exact test. The log-rank test was used to compare the rate of improvement. A significance level was set at a *p*-value of ≤ 0.050; *p*-values above this threshold were considered non-significant.

## Results

In this study, 75 patients were enrolled; 66 completed the study and were included in the final analysis (23 in the cyclosporine group and 43 in the SOC group) (Fig. 1).

Table 1 showed that there was no statistically significant difference between the studied groups regarding age, sex, respiratory rate, heart rate, white blood cells (WBC), and lymphocytes.

**Table 1:** Demographic characteristics of the study groups.

Variables	Cyclosporin group (Total = 23)	Standard group (Total = 43)	*p*-value
Age (years)	35.6 ± 14.4	32.7 ± 11.8	0.388
Sex			
Male	12 (52.2%)	18 (41.9%)	0.423
Female	11 (47.8%)	25 (58.1%)	
Respiratory rate (cycles/min)	18.8 ± 3.0	19.3 ± 2.9	0.535
Heart rate (beats/min)	94.7 ± 10.2	95.1 ± 9.2	0.859
WBC (× 10^3^/mL)	6.0 ± 1.1	5.9 ± 1.3	0.565
Lymphocytes (× 10^3^/mL)	1.02 ± 0.26	0.98 ± 0.31	0.561

Data presented as Mean ± SD or number (%).

**Fig. 2:**
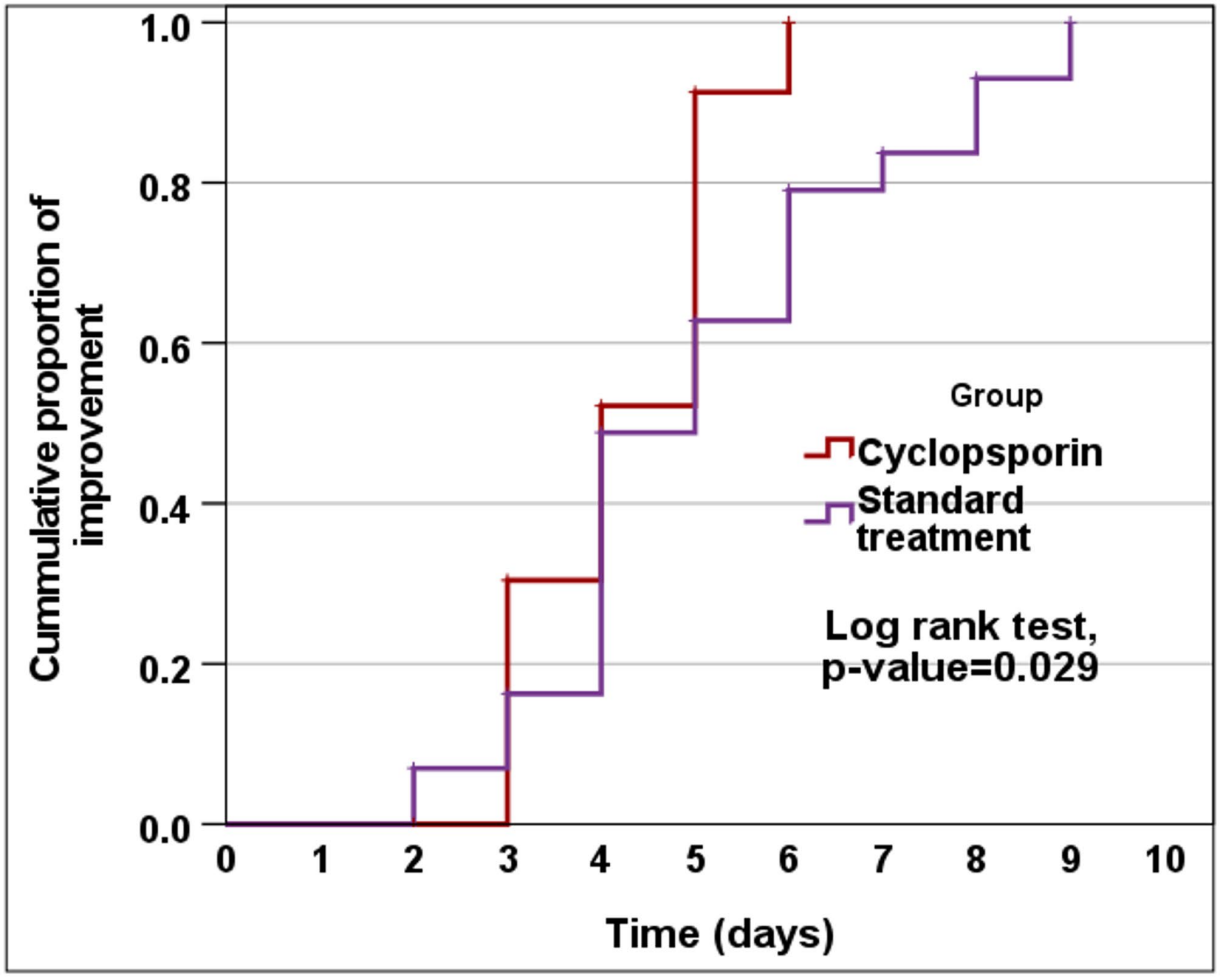
Kaplan–Meier curve for the rate of improvement between the study groups.

Figure 2 illustrated that the time to improvement was significantly shorter in the Cyclosporin group (4.3 ± 1.0 days) compared to the Standard group (5.1 ± 2.3 days), with a mean difference ± Standard error of − 0.8 ± 0.4 days (95% CI: − 1.6 to − 0.1, *p* = 0.025.

Oxygen supplementation in the Cyclosporin group was 7 (30.43%) compared to 12 (27.91%) in the Standard group, indicating no significant difference (RR = 1.00, 95% CI: 0.50–2.39, *p* = 0.828). Clinical status, assessed using the WHO seven-point ordinal scale, showed no statistically significant differences between the groups in terms of hospitalization, need for non-invasive ventilation, high-flow oxygen therapy, invasive mechanical ventilation, or mortality. None of the participants in either group required these interventions, and no adverse events of secondary fungal or bacterial infections occurred; therefore, statistical comparisons were not applicable. One case of diarrhea was graded as 3 over 28 days.

Table 2 analysis showed no significant baseline differences on enrollment between the study groups concerning baseline measurements of oxygen saturation, C-reactive protein (CRP), D-dimer, ferritin, and interleukin-6 (IL-6). However, the group receiving cyclosporine demonstrated significantly higher oxygen saturation levels at day 7, although levels at day 14 did not show significant differences. Additionally, the cyclosporine group exhibited significantly lower CRP and IL-6 levels, with reductions observed on both days 7 and 14. Similarly, D-dimer and ferritin levels were significantly reduced in the cyclosporine group at day 14, while changes at day 7 were not statistically significant.

**Table 2:** Laboratory findings between the study groups.

Findings	Values	Time points	Cyclosporin group (Total = 23)	Standard group (Total = 43)	*p*-value	Relative effect	
						Mean ± SE	95% CI
Oxygen saturation (%)	Level	Baseline	94.4 ± 2.1	94.7 ± 2.8	0.672	− 0.3 ± 0.7	− 1.6–1.0
		Day-7	97.4 ± 1.5	96.1 ± 1.7	0.003*	1.3 ± 0.4	0.5–2.2
		Day-14	98.5 ± 1.3	97.9 ± 1.4	0.072	0.7 ± 0.4	− 0.1–1.4
	Change	Day-7	3.0 ± 1.3	1.4 ± 1.6	< 0.001*	1.6 ± 0.4	0.8–2.4
		Day-14	4.1 ± 1.6	3.2 ± 2.0	0.055	0.9 ± 0.5	0.0–1.9
CRP (mg/L)	Level	Baseline	16.7 ± 12.8	14.6 ± 12.2	0.530	2.0 ± 3.2	− 4.4–8.4
		Day-7	4.7 ± 3.5	11.4 ± 10.3	0.004*	− 6.7 ± 2.2	− 11.2– − 2.3
		Day-14	3.3 ± 2.4	8.6 ± 7.8	0.002*	− 5.3 ± 1.7	− 8.6– − 2.0
	Change	Day-7	− 12.0 ± 11.2	− 3.2 ± 5.0	< 0.001*	− 8.8 ± 2.0	− 12.7– − 4.8
		Day-14	− 13.4 ± 12.0	− 6.0 ± 6.9	0.002*	− 7.3 ± 2.3	− 12.0– − 2.7
D-dimer (μg/L)	Level	Baseline	645.2 ± 84.4	652.9 ± 70.9	0.693	− 7.8 ± 19.6	− 46.9–31.4
		Day-7	584.4 ± 77.4	605.5 ± 69.8	0.264	− 21.1 ± 18.7	− 58.5–16.3
		Day-14	526.9 ± 67.3	582.7 ± 68.5	0.002*	− 55.8 ± 17.6	− 90.9– − 20.6
	Change	Day-7	− 60.7 ± 29.2	− 47.4 ± 32.7	0.106	− 13.3 ± 8.1	− 29.6–2.9
		Day-14	− 118.3 ± 54.5	− 70.2 ± 33.1	< 0.001*	− 48.0 ± 10.8	− 69.6– − 26.5
Ferritin (ng/mL)	Level	Baseline	178.9 ± 76.4	167.7 ± 61.4	0.522	11.1 ± 17.3	− 23.4–45.7
		Day-7	114.4 ± 55.4	126.4 ± 55.6	0.405	− 12.0 ± 14.3	− 40.7–16.6
		Day-14	85.6 ± 48.2	114.9 ± 52.1	0.029*	− 29.3 ± 13.1	− 55.5– − 3.1
	Change	Day-7	− 64.5 ± 58.9	− 41.3 ± 39.7	0.062	− 23.2 ± 12.2	− 47.5–1.2
		Day-14	− 93.3 ± 62.8	− 52.9 ± 37.0	0.002*	− 40.4 ± 12.3	− 64.9– − 15.9
IL-6 (pg/mL)	Level	Baseline	18.2 ± 6.5	17.3 ± 5.0	0.497	1.0 ± 1.4	− 1.9–3.8
		Day-7	9.7 ± 2.8	13.3 ± 4.9	0.002*	− 3.6 ± 1.1	− 5.8– − 1.4
		Day-14	6.1 ± 2.0	10.8 ± 4.9	< 0.001*	− 4.7 ± 1.1	− 6.9– − 2.6
	Change	Day-7	− 8.5 ± 4.5	− 3.9 ± 2.3	< 0.001*	− 4.6 ± 0.8	− 6.2– − 2.9
		Day-14	− 12.2 ± 6.1	− 6.5 ± 2.5	< 0.001*	− 5.7 ± 1.1	− 7.8– − 3.6

Data presented as Mean ± SD unless mentioned otherwise. Independent t-test. Change = Day 7 or 14 value –baseline, negative values indicate reduction. *Significant. Relative effect: Effect in the cyclosporin group relative to that in the standard treatment group. SE: Standard error. CI: Confidence interval.

## Discussion

The study involved 66 patients who were randomly assigned to two groups to evaluate the clinical outcomes and biochemical markers in COVID-19 patients with moderate or severe disease. One group was treated with oral cyclosporine capsules at a dosage of 6 mg/kg/day, while the other group received a standard treatment regimen. Our findings indicated no significant differences in clinical outcomes between the two groups, except for a one-day shorter time to improvement. However, the cyclosporine group demonstrated a significantly greater reduction in D-dimer, ferritin, and CRP levels. Furthermore, cyclosporine was found to be safe, as no adverse drug events were reported among the participants in the study.

COVID-19 outcomes are linked to increased systemic cytokines and laboratory abnormalities, indicating hyperinflammation and tissue damage, such as elevated CRP and d-dimer levels, resembling cytokine storm disorders^45^. Elevated nonspecific inflammatory markers, like ferritin and C-reactive protein (CRP) levels, were correlated with cytokine storms^46^. Other literature illustrated that COVID-19-related systemic inflammation is considered part of the hyperferritinemic syndromes^47^. High serum IL-6 levels independently and strongly predicted COVID-19 patients’ survival^48,49^.

In our study, there were no deaths or the need for mechanical ventilation. According to a 2021 review article, the global death rate is 1%, with the lowest number of deaths occurring in Africa^50^. Another study showed that CsA enhances outcomes and reduces mortality (by 35%) in COVID-19 patients when administered as an adjunct to steroid therapy^51^. A retrospective study indicated that cyclosporine A (CsA) may reduce mortality in patients with severe COVID-19^52^.In a pilot randomized clinical trial, CsA showed a trend toward increasing the proportion of COVID-19 patients without interstitial lung disease at 3 months, including both those who did and did not require IMV^53^. On the other hand, a study demonstrated no significant differences in adverse events or the effect on invasive mechanical ventilation when comparing CsA in addition to standard care versus standard care alone^53^.

The cyclosporin effect may help prevent lung damage from hyperinflammation, inhibit viral replication, and also provide a cost-effective solution^54^. A retrospective cohort study showed that patients with rheumatic disorders who are exhibiting severe disease activity and a chronic SARS-CoV-2 infection may benefit from CsA^55^.

We measured oxygen saturation using pulse oximetry, which is crucial for detecting hypoxemia but can sometimes be inaccurate. Factors such as skin pigmentation, temperature, and tobacco use can affect readings. Nevertheless, the Food and Drug Administration (FDA) recommends at-home use of pulse oximeters, noting they can reliably estimate blood oxygen saturation. To illustrate, a SpO2 of 90% typically reflects an arterial oxygen saturation (SaO2) of 86–94%. Incorporating pulse oximetry into your health routine is important^56^.

Ivermectin and azithromycin, with or without favipiravir, were used as standard treatments by the MOH protocol in 2022. Treatment with ivermectin significantly shortened the time to viral clearance compared to the control group^57^. However, a meta-analysis conducted in 2025 found it ineffective for treatment or prophylaxis^58^.The Cochrane review found that azithromycin neither reduced mortality nor improved clinical outcomes in patients with COVID-19^59^.

On patient follow-up regarding the safety of the drug, no severe or life-threatening adverse drug events were recorded in either group, except for 1 case suffering diarrhea grade 4, and none of the patients suffered an invasive infection. On the other hand, another study demonstrated that CAS was safe but increased liver and kidney enzymes in some patients. We speculate that due to differences in the dose, they used a higher dose than in our study (9 mg/kg/day for 6 days)^60^; however, we used 6 mg/kg/day CAS in a divided dose for 7 days, and most patients improved after an average of 4 days. Another study revealed that cyclosporin has a narrow therapeutic range, and the side effects are reversible with decreasing doses^61^.

Our study has limitations. A formal sample size calculation was not conducted because the trial was registered in early 2021, and there was no prior data available to guide the estimation of power. However, we were able to achieve the target enrollment specified in the updated protocol. Additionally, the limited selection of patients—primarily chosen for safety reasons—restricts the generalizability of our findings. We did not use chest CT for follow-up visits unless patients showed clinical deterioration, which was in line with WHO and American College of Radiology guidelines. A WHO rapid device guide on June 11, 2020, noted that CT imaging findings from hospitalization were associated with adverse clinical outcomes, though the evidence was limited^62^. Lastly, the lack of direct comparisons with other immunomodulators hinders our ability to contextualize the therapeutic role of cyclosporine A in relation to alternative treatment options.

Future research should involve larger sample sizes, different dosing strategies, and direct comparisons with other immunomodulators, such as IL-6 inhibitors. It is also crucial to focus on high-risk patients, particularly those experiencing a cytokine storm who require mechanical ventilation, to better define the role of cyclosporine A in treating COVID-19.

## Conclusion

Cyclosporine did not significantly improve seven ordinal scale outcomes. However, it was associated with a shorter time to clinical improvement and favorable modulation of inflammatory markers in patients with COVID-19 and cytokine storm, without major safety concerns, and Cyclosporine did not significantly improve seven ordinal scale outcomes. However, it was linked to a shorter time to clinical improvement and favorable changes in inflammatory markers in patients with COVID-19 who experienced cytokine storms. There were no major safety concerns, suggesting that cyclosporine may be a valuable adjunct therapy for managing hyperinflammation in patients with moderate to severe COVID-19. This is supported by its impact on markers such as D-dimer, ferritin, IL-6, and CRP. Additionally, its potential role in controlling cytokine storms, along with its availability in capsule form and ease of use, makes it a potentially accessible therapeutic option worldwide.

## Data Availability

The datasets generated and/or analysed during the current study are not publicly available due to privacy or ethical restrictions, but are available from the corresponding author on reasonable request.
